# Veterinary Technicians and Occupational Burnout

**DOI:** 10.3389/fvets.2020.00328

**Published:** 2020-06-12

**Authors:** Lori R. Kogan, Jean E. Wallace, Regina Schoenfeld-Tacher, Peter W. Hellyer, Madeline Richards

**Affiliations:** ^1^Department of Clinical Sciences, College of Veterinary Medicine and Biomedical Sciences, Colorado State University, Fort Collins, CO, United States; ^2^Department of Sociology, Faculty of Arts, University of Calgary, Calgary, AB, Canada; ^3^Department of Molecular Biomedical Sciences, College of Veterinary Medicine, North Carolina State University, Raleigh, NC, United States; ^4^Department of Biomedical Sciences, Colorado State University, Fort Collins, CO, United States

**Keywords:** burnout, veterinary technicians, occupational stress, Maslach Burnout Inventory, Stanford Professional Fulfillment Index

## Abstract

Burnout and compassion fatigue are common conditions affecting health care providers. Unique occupational conditions in veterinary medicine make technicians especially susceptible to burnout. A total of 1,642 practicing veterinary technicians completed an anonymous online survey comprised of demographic questions, and two tools to assess burnout: the Maslach Burnout Inventory-General Survey (MBI-GS) and the Stanford Professional Fulfillment Index (PFI). Over half of participants (862/1479, 58.3%) had EE scores over the 3.0 threshold for burnout. On the PFI, the total score for the 10 burnout questions was *x* = 1.54 (*SD* = 0.75), which is above the 1.33 cutoff for burnout. The mean score of 2.26 (*SD* = 0.81) on the professional fulfillment scale is also indicative of burnout. The relationship between enabling resources and scores on each MBI-GS scale was analyzed. Schedule control was the most significant predictor of lower EE scores. The perception of adding value to the practice was associated with lower scores on the CY scale and higher scores on the PE scale. Given the correlation between burnout and environmental factors, veterinary practices are encouraged to explore non-monetary mechanisms for enhancing job satisfaction. This includes giving technicians greater control over their schedules, recognizing their contributions to the team, and providing opportunities for professional development. From a morale standpoint, destigmatizing the dirty work done by technicians can also help combat burnout among veterinary technicians.

## Introduction

Research pertaining to health care providers' burnout and compassion fatigue is plentiful within human medical fields. Although exact figures of the percentage of medical professionals suffering from burnout vary, most estimates exceed 50%, presenting a public health concern that impacts not only the professional, but patients, coworkers, family members and health care organizations (1–6). Although fewer studies have been conducted pertaining to veterinary professionals, they have reached similar conclusions; namely that burnout and compassion fatigue are common among this population and can lead to serious negative physical, and psychological impact (7).

Compassion fatigue can be defined as exhaustion due to the demands of being empathic and helpful to those who are suffering (8). It is often the result of witnessing trauma or being involved in another's painful experience and can lead to burnout—a psychological syndrome comprised of emotional exhaustion, depersonalization and a sense of reduced personal accomplishment (9, 10). Emotional exhaustion relates to the depletion of one's emotional resources; depersonalization refers to cynical, callous or detached attitudes toward the job, clients or patients; and lack of personal accomplishment can be defined as a negative self-appraisal of incompetence and ineffectuality (10, 11). Burnout is typically the result of both external and internal stressors (12) and often measured across three dimensions: exhaustion, cynicism, and a sense of inefficacy (13). The Maslach Burnout Inventory used in this study is the most commonly used instrument to assess burnout and consists of these three subscales (14).

Work factors found to contribute to physician burnout include excessive workloads, long working hours, frequent on-call duties, and excessive time spent on documentation/paperwork (5, 6). In addition, a loss of autonomy and decreased control over the work environment have been found to be common factors leading to burnout (15). Factors found to contribute to burnout in veterinarians include excessive workload and work hours, on-call duties, limited resources, workplace conflicts, and the unique challenges that come with euthanasia (7, 16–20). Additional stressors for veterinary professionals include unrealistic expectations from pet owners, situations where they need to balance the affordability of treatment with the provision of high quality care, and low income paired with high debt load (12, 21–24).

More recently, studies have explored the role of enabling resources that may enhance worker engagement, meaningful work and well-being while reducing feelings of burnout and compassion fatigue (25–27). Work factors that appear to mitigate burnout, for physicians and veterinarians alike, include schedule control, opportunities for professional development and the use of skills and knowledge, the ability to develop and use their skills, respect from colleagues, and a feeling of satisfaction with one's position/job (4, 16, 18). Cake et al. (25) proposed that animal care workers' opportunities for self-actualization may result from applying their specialized skills and knowledge to meeting the challenges of their work and that self-actualizing work may enhance their engagement, feelings of accomplishment and personal growth as well contribute to a sense of being involved in meaningful work. Work environments that promote these positive responses may foster resilience and well-being for those encountering highly stressful work situations (10, 26). Personal characteristics associated with physician burnout include being self-critical, engaging in unhelpful coping strategies, sleep deprivation, over commitment, perfectionism, poor work–life balance, and an inadequate support system outside the work environment (15, 28). Similar results have been found among veterinarians (16) with younger, female, and single veterinarians most at risk for psychological distress (23). Dawson (29) has suggested that personality characteristics might even play a larger role than occupational factors in predicting workplace stress among veterinary professionals.

We examine four clusters of enabling resources, plus financial compensation, that may offer veterinary technicians a sense of purpose, meaning and personal growth in their work and explore how these factors relate to each of the three dimensions of burnout. Because these factors may impact burnout differently, they were each assessed separately. Schedule control refers to the flexibility and autonomy technicians have in regards to their work schedules. Schedule control includes flexibility of one's schedule, as well as control over days/hours, time at work, and length of shifts.

Having a sense of autonomy (decision latitude) is a widely recognized resource that is positively related to well-being (16, 30). Using skills and knowledge contributes to a sense of accomplishment, personal growth and engagement. Technicians who have the opportunity to solve complex problems and contribute significantly to animal care will feel their work is more meaningful and fulfilling (27). Opportunities for learning and success are key to enhancing a sense of accomplishment and personal growth that result in meaningful engaging work (25, 26). Respect from colleagues can foster a sense of community, trust and belonging in the workplace.

Veterinary professionals' high rates of stress, burnout, and emotional exhaustion (31) are especially alarming given the fact that male veterinarians' suicide rates are 2.1 times as high and female veterinarians are 3.5 times as high as the general U.S. population (32). They are also more likely to die from suicide than other health care professionals (33). Perhaps even more staggering are the rates of suicidal ideation (seriously thinking of taking one's own life) among veterinarians. Studies from the United Kingdom (U.K.), United States (U.S), Australia and Canada report that ~20% of all veterinarians have had suicidal thoughts in the past year, compared to ~3% in the general adult population (24). Most veterinary professionals' mental health studies, however, have focused on veterinarians, with very little research on mental health, burnout and compassion fatigue among veterinary technicians who work alongside veterinarians in the same work settings where they face the same potential stressors (20, 34–36).

Veterinary technicians are a critical (and growing) component of successful veterinary practices. The field of veterinary technicians is still relatively young, with the first class graduating from an animal technician program in 1963 and the first American Veterinary Medical Association (AVMA) accredited program created in 1973 (37). Growing quickly, U.S. veterinary technicians now number over 109,000 and the U.S. Bureau estimates an anticipated 19% job growth from 2018 to 2028—much faster than average. The median pay for a veterinary technician in the U.S. in 2018 was $34,420/year or $16.55/h (38). It should also be noted that this job is historically and currently predominantly held by women; recent statistics show 95% of veterinary technicians are women (39). Given women's higher propensity to suffer from burnout, compassion fatigue and suicide compared to men (16, 26), members of this female-dominated occupation may be particularly at risk.

Typical veterinary technician duties include: collecting and recording medical histories, providing nursing care and emergency first aid to recovering or injured animals, administering anesthesia, preparing both the patient and equipment for surgery as well as monitoring the animal during surgery, administering medications, vaccines, and treatments as prescribed by the veterinarian, performing diagnostics like radiographs and laboratory tests, and restraining animals during exams and other procedures (38). Arguably one of the most important yet often challenging part of technicians' responsibilities involves client communication. The technician is often the liaison between the client and the veterinarian. To maximize veterinarians' time, technicians often collect client and patient information and answer clients' questions. A survey by The National Association of Veterinary Technicians (39) found that 79% of technicians report that as part of their job, they instruct owners on how to administer medications, 71% instruct owners on how to properly care for pets, and 56% discuss with owners how to manage their pet's pain. The tasks and communication performed by veterinary technicians is vital for the successful operation of veterinary practices. To this point, 68% of clinics schedule technician-only appointments that do not require veterinarian assistance (39).

Perhaps it is not surprising, given the technicians' job duties, that preliminary studies indicate that veterinary technicians are also at high risk for occupational stress and burnout (30, 40). One study found factors predictive of increased burnout for technicians include work load, job demands, exposure to euthanasia and contact with clients (34). Job control and social support were found to be negatively associated with their burnout levels (34). Animal technicians also have higher than average rates of turnover (39) compared to other occupations, which is another indicator of high levels of job stress and burnout. Based on the results of the NAVTA 2016 survey (39), it was concluded that turnover may result when technicians do not feel they are part of the team or that they are not working toward a common purpose (41).

As veterinary technicians continue to grow in numbers and assume more responsibilities, it is critical to better understand factors that predict burnout as well as potential mitigating circumstances. The current study was designed to identify factors that may improve veterinary technicians' work experience by mitigating burnout. As noted above, very little research has examined the extent to which veterinary technicians' work is intellectually fulfilling and rewarding or how they apply and acquire a variety of skills that they can use in complex problem solving (30).

The positive work characteristics examined in this study reflect enabling resources that may facilitate well-being and resilience among veterinary technicians (16, 24, 25). We examine four clusters of enabling resources, plus financial compensation, that may offer veterinary technicians a sense of purpose, meaning and personal growth in their work and explore how these factors relate to each of the three dimensions of burnout. Schedule control refers to the flexibility and autonomy technicians have in regards to their work schedules. Having a sense of autonomy (decision latitude) is a widely recognized resource that is positively related to well-being (16, 30). Using skills and knowledge contributes to a sense of accomplishment, personal growth and engagement. Technicians who have the opportunity to solve complex problems and contribute significantly to animal care will feel their work is more meaningful and fulfilling (27). Opportunities for learning and success are key to enhancing a sense of accomplishment and personal growth that result in meaningful engaging work (25, 26). Respect from colleagues can foster a sense of community, trust and belonging in the workplace. Those who feel connected to others may feel more supported and more likely to seek and offer assistance to one another (25).

## Materials and Methods

An online, anonymous, cross-sectional survey was developed using Qualtrics (Qualtrics, Inc.; Provo, UT, USA). The survey was designed, reviewed, and tested by the co-investigators and their colleagues. A portion of the survey consisted of the Maslach Burnout Inventory-General Survey (MBI-GS). The MBI-GS consists of 16 items divided into three scales with reported reliabilities ranging as follows: emotional exhaustion (EE) (five items; α = 0.84–0.90), cynicism (CY) (four items; α = 0.74–0.84) and professional efficacy (PE) (seven items; α = 0.70–0.78) (42). The questions are scored using a seven level frequency scale from “never” to “daily.” The MBI-GS was not designed to combine the scales for a single burnout scale but to assess each of the three scales separately. Examples of questions include, “I feel emotionally drained from my work” (EE); “I have become less interested in my work since I started this job” (CY); and “I can effectively solve the problems that arise in my work” (PE, reverse coded). It has been suggested that scoring can be done by using each third of the potential range of scores to indicate “low,” “average,” and “high” scores on burnout (43).

Many studies dichotomize results into burnout/no burnout but there is no accepted standard definition or criterion (44). Determining burnout has been done in several ways with the most common methods including a combination of high EE, high CY and low PE; high EE and/or high CY, and high levels in EE subscale only. According to the Maslach Burnout Inventory manual (43) individuals with scores of ≥3.2 on the EE subscale, ≥2.6 on the CY subscale, or ≤ 3.8 on the PE subscale can be classified as having high burnout levels for that particular scale. Other studies (45, 46) have defined severe burnout as a mean > 3.0 for EE.

Additionally, the survey included the Stanford Professional Fulfillment Index (PFI) (47). This tool was recently developed, so does not have the decades of supportive research that accompanies the MBI, yet we felt it has the potential to accurately assess our population's burnout and fulfillment levels. We included the PFI to assess its validity for veterinary technicians. We correlated scores on the PFI subscales (Professional Fulfillment, Work Exhaustion, and Interpersonal Disengagement) with the scores of similar subscales on the MBI (Professional Efficacy, Emotional Exhaustion, and Cynicism, respectively). The PFI is a 16-item survey with three scales: two scales measure burnout in terms of work exhaustion (four questions) and interpersonal disengagement (six questions); and one scale that measures professional fulfillment (six questions). Response options are on a five-point Likert scale (“not at all true” to “completely true”) for professional fulfillment items and “not at all” to “extremely” for work exhaustion and interpersonal disengagement items.

Items are scored 0–4 with each dimension treated as a continuous variable. Scale scores are calculated by averaging the item scores of all the items within the corresponding scale. Higher scores on the professional fulfillment scale are viewed more favorably while higher scores on the work exhaustion or interpersonal disengagement scales are less favorable. Dichotomous burnout categories are determined from the average item score of all 10 burnout items (work exhaustion and interpersonal disengagement), using a cut-point of 1.33. Dichotomous professional fulfillment is recommended at an average item score cut-point of >3.0 (47).

Reported test-retest reliability estimates are 0.82 for professional fulfillment (α = 0.91), 0.80 for work exhaustion (α = 0.86), 0.71 for interpersonal disengagement (α = 0.92), and 0.80 for overall burnout (α = 0.92) (47). Trockel (47) reported a correlation between the PFI work exhaustion subscale score and MBI emotional exhaustion subscale score of 0.72; a correlation between PFI interpersonal disengagement score and MBI cynicism subscale score of 0.59; and a correlation between the PFI Professional Fulfillment score and MBI Professional Efficacy subscale score of 0.46.

To assess the validity of using the Stanford Professional Fulfillment Index (PFI) for veterinary technicians, scores on the PFI scales Professional Fulfillment, Work Exhaustion, and Interpersonal Disengagement were correlated with the scores of similar scales on the MBI.

Other elements of the survey included demographic questions: sex, age, and country of residence. Participants were also asked questions related to their work setting, whether they were in a supervisor/management role, years working within veterinary medicine, and years working as a veterinary technician. The work-related questions included a screening question asking if they were or were not a credentialed veterinary technician. Only those who reported they were currently a credentialed veterinary technician were included in further analysis.

Participants were asked to report their current satisfaction on a 5-point Likert scale with several enabling resources available to them in their employment setting. Five sets of enabling resources were measured that include: financial compensation, schedule control, using skills and knowledge, respect from colleagues, and learning and success. Anchor choices included 1–very unsatisfied and 5–very satisfied. They were also asked to indicate how important each of these enabling resources are to them, using a 5-point Likert scale with anchor choices including 1–very unimportant and 5–very important. **Table 3** provides a description of each of the items.

Lastly, to assess one possible intervention for veterinary technicians' burnout, additional education and credentialing questions were asked. These items are included for descriptive purposes and their responses are not included in the current analysis.

The survey was pilot tested by ten individuals for ambiguity and/or potentially missing or inappropriate response options, with revisions made based on the results of the pilot testing. The final survey and study design were approved by the Colorado State University Institutional Review Board (IRB # 086-19H). Survey respondents were recruited through social media platforms (Facebook and Twitter) between November 2018 and February 2019.

Data were analyzed using SPSS (IBM SPSS; version 25). First, descriptive statistics were used to characterize participants (see Tables 1, 2). After testing that the assumptions of linear regression had been met, linear regression was used to explore the function of enabling resources in relation to the three MBI components (Table 3). Lastly, Pearson's correlation was used to compare scores between the subscales of the MBI to those of the PFI.

**Table 1:** Demographic data from all participants.

**Demographics**						
Country (*n* = 1,642)	Canada	United States	Other			
	170 (10.4)	1,443 (87.9%)	29 (1.8%)			
Years in the field (*n* = 1,642)	<3 years	3–5 years	6–10 years	11–15 years	16–20 years	More than 20 years
	131 (8.0%)	318 (19.4%)	418 (25.5%)	314 (19.1%)	200 (12.2%)	261 (15.9%)
Years have been a Credentialed Veterinary Technician (*n* = 1,551)	<3 years	3–5 years	6–10 years	11–15 years	16–20 years	More than 20 years
	346 (22.3%)	329 (21.2%)	382 (24.6%)	242 (15.6%)	139 (9.0%)	113 (7.3%)
Gender (*n* = 1,549)	Male	Female	Other/NA			
	46 (3.0%)	1,495 (96.5%)	8 (0.5%)			
Do you have a supervisory or management position? (*n* = 1,552)	No	Yes				
	1,038 (66.9%)	514 (33.2%)				
Years at current place of employment (*n* = 1,552)	<3 years	3–5 years	6–10 years	11–15 years	16–20 years	More than 20 years
	663 (42.7%)	411 (26.5%)	244 (15.7%)	117 (7.5%)	65 (4.2%)	52 (3.4%)

**Table 2:** The hours worked per week and the current pay of the participants.

**Work Figures**											
How many hours/week? (*n* = 1,552)	<10	10–20	21–30	31–40	41–50	51–60	More than 60				
	16 (1.0%)	23 (1.5%)	79 (5.1%)	711 (45.8%)	624 (40.2%)	80 (5.2%)	19 (1.2%)				
											
Current pay (*n*= 1,548)	Federal	$10/h	$10–15/h	$16–20/h	$21–25/h	$26–30/h	$31–35/h	$36–40/h	$41–45/h	$46–50/h	More than $50
	min wage/h										
	3 (0.2%)	8 (0.5%)	222 (14.3%)	690 (44.6%)	364 (23.5%)	156 (10.1%)	57 (3.7%)	23 (1.5%)	10 (0.6%)	7 (0.5%)	8 (0.5%)

**Table 3:** Enabling resources as they relate to each component of the Maslach Burnout Inventory.

**Enabling resources**	**Exhaustion** ***Beta*,** ***p*-value, 95% CI**	**Cynicism** ***Beta*, *p*-value, 95% CI**	**Professional efficacy** ***Beta*, *p*-value, 95% CI**
**Financial Rewards**			
Current pay/salary	−0.03, 0.24 (−0.10, 0.03)	−0.03, 0.21 (−0.11, 0.03)	**−0.05, 0.05 (−0.09, 0.00)**
**Schedule Control**			
Flexibility of schedule	−0.01, 0.88 (−0.9, 0.8)	0.01, 0.78 (−0.08, 0.10)	**−0.07, 0.05 (−0.11, 0.00)**
Control over schedule (days/hours)	0.04, 0.30 (**–**0.5, 0.14)	**–**0.01, 0.80 (**–**0.11, 0.08)	**0.08, 0.04 (0.00, 0.12)**
Control over time at work	**−0.20**, ** <0.001 (−0.34**, **−0.17)**	−0.01, 0.67 (−0.11, 0.07)	−0.03, 0.38 (−0.09, 0.03)
Control over length of shifts	–**0.20**, ** <0.001 (**–**0.35**, –**0.20)**	–**0.10, 0.00 (**–**0.22**, –**0.06)**	0.03, 0.39 (−0.03, 0.07)
**Using Skills and Knowledge**			
Feeling of adding value to the practice/place of employment	**−0.12**, ** <0.001 (−0.25**, **−0.10)**	**−0.30, 0.00 (−0.52**, **−0.34)**	**0.40**, **<** **0.001 (0.30, 0.42)**
Proper utilization of professional skills	0.05, 0.08 (−0.01, 0.12)	−0.02, 0.44 (−0.10, 0.04)	0.05, 0.12 (−0.01, 0.08)
Feeling that the veterinarians you work with are aware of your skills	0.04, 0.30 (−0.04, 0.13)	0.06, 0.10 (−0.01, 0.16)	**0.08, 0.02 (0.01, 0.12)**
**Learning and Success**			
Opportunity for career mobility	**−0.15, 0.00 (−0.30**, **−0.12)**	**−0.15, 0.00 (−0.31**, **−0.13)**	0.01, 0.88 (**–**0.06, 0.06)
Opportunity for self-improvement and/or professional development	**−0.10, 0.00 (−0.21**, **−0.04)**	**−0.14, 0.00 (−0.30**, **−0.11)**	0.03, 0.34 (−0.03, 0.09)
Opportunity to affect change when something that could be improved/changed	**–**0.06, 0.07 (**–**0.14, 0.01)	**–**0.04, 0.14 (**–**0.13, 0.02)	**0.09**, ** <0.001 (0.02, 0.12)**
**Respect From Colleagues**			
Respect from veterinarians at work	**−0.07, 0.03 (−0.17**, **−0.01)**	**−0.11, 0.00 (−0.22**, **−0.06)**	−0.00, 0.92 (−0.06, 0.05)
Respect from credentialed veterinary technicians	**0.08, 0.01 (0.03, 0.20)**	0.03, 0.40 (−0.05, 0.12)	−0.00, 0.91 (−0.06, 0.05)
Respect from other support staff	**−0.10**, ** <0.001 (−0.20**, **−0.05)**	−0.04, 0.14 (−0.14, 0.02)	−0.02, 0.51 (−0.07, 0.03)

*Bolded items are significantly related to that component*.

## Results

A total of 1,642 responses were obtained from credentialed veterinary technicians, of which 1,443 (87.9%) reported living in the United States, 170 (10.4%) in Canada, and 29 (1.8%) in other countries. This sample was primarily female (1495, 96.5%).

When asked how long they had been working in the veterinary field, almost half (775, 47.2%) of the responders reported having worked in the field for more than 10 years, and the other half (867, 52.8%) have been a credentialed veterinary technician for 10 years or less. Pertaining to their current work setting, the largest number reported currently working in a companion/small animal practice (668; 43.0%), specialty hospital (270, 17.4%) or emergency hospital (244, 15.7%) and have worked in their current setting for <3 years (663; 42.7%). The majority reported working between 31 and 40 h a week (711, 45.8%) or 41–50 h a week (624, 40.2%) and not having a supervisory or management position (1038, 66.9%). When asked about salary, about half of respondents reported earning between $16 and 20/h (690, 44.6%). Demographic data on survey respondents are summarized in Table 1.

### MBI Scale Scores

The mean summation scores for each of the MBI scales were calculated. Results were EE scale: *X* = 17.35 (*SD* = 7.2); CY scale: *X* = 12.69 (*SD* = 7.9); and PE scale: *x* = 28.97 (*SD* = 5.68). The mean average scores were EE scale: *x* = 3.47 (*SD* = 1.44); CY scale: *X* = 2.55 (*SD* = 1.58); and PE scale: *x* = 4.82 (*SD* = 0.95). Cronbach's alpha for the EE scale for this sample was 0.91, 0.86 for the CY scale and 0.76 for the PE scale. Using the cut off scores of ≥3.2 on the EE scale, ≥2.6 on the CY scale, or ≤ 3.8 on the PE scale (43), these results place the participants at high levels of burnout. Looking at the mean EE scale cutoff score of 3.0, 862/1,479 (58.3%) participants scored above the burnout threshold.

### PFI Scores

The mean total scores for each of the PFI scales were calculated: Work Exhaustion scale: *X* = 1.93 (*SD* = 0.90); Interpersonal Disengagement scale: *X* = 1.28 (*SD* = 0.77); and Professional Fulfillment scale: *X* = 2.26 (*SD* = 0.81). The total score for the 10 burnout questions (Work Exhaustion and Interpersonal Disengagement) was *X* = 1.54 (*SD* = 0.75). Cronbach's alpha for the Professional Fulfillment scale for this sample was 0.86, 0.86 for the Work Exhaustion scale, 0.87 for the Interpersonal Disengagement scale, and 0.90 for the total Burnout score (combined scales of Work Exhaustion and Interpersonal Disengagement).

Using the suggested dichotomous burnout categories of all 10 burnout items (work exhaustion and interpersonal disengagement) of 1.33, the results of 1.54 suggest that this population exceeds the cutoff for being determined as burnout. Additionally, the mean score of 2.26 of this sample falls under the cutoff point of >3.0 for the Professional Fulfillment scale.

### Correlations Between the MBI and PFI Scales

Pearson's correlations were conducted on the corresponding scales of the MBI and the PFI. These resulted in the following: MBI Work Exhaustion and PFI EE: *r* = 0.80 (*p* < 0.001; 95% CI: 0.78, 0.81); MBI CY and PFI Interpersonal Disengagement: *r* = 0.61 (*p* < 0.001; 95% CI: 0.59, 0.64); and MBI PE and PFI Professional Fulfillment: *r* = 0.60 (*p* < 0.001; 95% CI: 0.58, 0.63).

### Enabling Resources and Burnout

Linear regression was used to assess the relationships between the enabling resources and the three components of the MBI (Table 3).

#### Emotional Exhaustion

The overall regression model for the MBI EE scale was significant, *F*_(14,1374)_ = 53.67, *p* < 0.001, *R*^2^ = 0.35. Eight factors were unique significant predictors of participants' EE scale scores. The most important enabling resources reflect schedule control (over time at work and length of shifts) and learning and success (related to career mobility and self/professional development) and skills and knowledge (feeling of adding value to the practice). Respect from other veterinary technicians is associated with greater, rather than reduced, emotional exhaustion.

#### Cynicism

The overall regression model for the MBI CY scale was significant, *F*_(14,1374)_ = 67.75, *p* < 0.001, *R*^2^ = 0.41. Five factors were unique significant predictors of participants' CY scale scores. Using skills and knowledge in terms of adding value to veterinary practice is by far the strongest predictor of cynicism. Learning and success also appears relevant in terms of career mobility and self-improvement or professional development that are negatively related to cynicism as well as respect from veterinarians.

#### Personal Efficacy

The overall regression model for the MBI PE scale was significant, *F*_(14,1374)_ = 39.83, *p* < 0.001, *R*^2^ = 0.29. Six factors were unique significant predictors of participants' professional efficacy scale scores. Similar to the findings for cynicism, using skills and knowledge by significantly contributing to veterinary practice is by far the strongest predictor of professional efficacy. Surprisingly, current pay and schedule flexibility reduce technicians' sense of efficacy rather than enhance it.

## Discussion

This study set out to explore burnout amongst veterinary technicians and the extent to which certain enabling resources might mitigate feelings of burnout. Participants in our survey reported high levels of burnout across all three dimensions of the MBI: high emotional exhaustion, high cynicism, and low professional efficacy. These results are corroborated by their high PFI scores on work exhaustion and interpersonal engagement and low scores on professional fulfillment. These findings signal that burnout is indeed a concern for this group of animal health care providers.

While many of the respondents have been working in the veterinary field for more than 10 years, it is interesting to note that the majority have acquired their credentials as a veterinary technician in the last 10 years. This suggests that many veterinary technicians start out in non-credentialed veterinary care positions before they train to become a licensed veterinary technician. Despite their extensive experience in the veterinary field, many technicians have only a few years of experience at their current place of employment. This pattern of results is consistent with other studies that found veterinary technicians have higher than average rates of turnover (39), which is another indicator of high rates of job stress and burnout.

This study also explored whether work-related enabling resources might mitigate against burnout. Overall, our findings suggest that these resources can be beneficial in reducing the different components of burnout. Several noteworthy findings are discussed in greater detail below.

First, greater control over work time and length of shifts are key in reducing emotional exhaustion. Having control over one's schedule may be particularly important for members of this occupation for two reasons. One is that veterinary technicians are an occupational group that generally has little autonomy and discretion over other aspects of their work as their primary function is to assist and follow instructions from veterinarians in performing patient care and technical tasks (31). Job control has a long history in the stress and burnout literature where it is argued that discretion over one's work can make achieving work goals more predictable and reduce anxiety associated with feeling overwhelmed and uncertain about work demands (48). It appears that having a sense of control over time spent at work is important in reducing emotional exhaustion for veterinary technicians. Furthermore, for this predominantly female occupation, control over the amount of time and length of shifts may be critical in minimizing work interference on their family life. Time pressures and overload at work may contribute to conflicts at home, work-family conflict and overall emotional exhaustion (16, 49, 50). Mastenbroek et al. (50) found that work-home interference was the main predictor of emotional exhaustion for both female and male veterinarians and identified work-family interferences as an obvious target for employer intervention. Exercising control over one's work schedule can be one strategy that may help veterinary technicians cope more effectively with heavy workloads and successfully combining work with family life.

Second, using one's skills and knowledge, particularly in terms of making a valuable contribution to practicing veterinary medicine, is key in mitigating all three components of burnout, especially in terms of reducing cynicism and enhancing a sense of professional efficacy. Using one's specialized skills and knowledge in contributing to the care of animals is important in making work meaningful (25, 27). Recognition for individuals' efforts and contributions to the shared goal of providing the best animal care is one strategy that veterinary practices may employee to support technicians sense of value to the animal health care team.

Much of the animal nursing carried out by veterinary technicians may be classified as physically dirty work (51, 52). Veterinary technicians' care work often involves contact with various forms of bodily fluids and wastes, exposure to disease and death, and the disposal of dead animals (52). The perceptions surrounding this type of work is often transferred to those who perform it and dirty workers are usually aware of the stigma and disregard associated with their job (51). If dirty workers are able to reframe the work they do as meaningful and important, it may reduce the harmful stigmatizing effects it has on their identity and well-being. In the case of veterinary technicians, our results suggest that if they feel that using their skills, which likely involves performing dirty tasks, translates into better care for the animals being treated in their care, they are significantly less emotionally exhausted, less cynical and feel more professional efficacy in their work. Wallace (27) found that veterinarians who felt their work was fulfilling and meaningful had increased feelings of well-being. Future research might explore how workers successfully manage stigma associated with dirty work so that they reframe their work as meaningful, important and less stressful.

Third, opportunities for learning and success, particularly in terms of self-improvement, professional development and career mobility are important in mitigating emotional exhaustion and cynicism and enhancing professional efficacy. These resources are key to self actualization and achieving occupational goals (25) as well as promoting work engagement (50). Providing opportunities for professional development and career success appears to be an important route to enhancing veterinary technicians' well-being. One way employers can support these opportunities is to grant time off for technicians to take continuing education courses and reimburse them for continuing education fees. In addition, employers might discuss and plan professional development and career goals with technicians to acknowledge their value to the animal health care team and promote commitment from both parties to a long-term employment relationship.

Fourth, respect from colleagues reduces emotional exhaustion and cynicism. In veterinary practice, veterinary technicians can be considered as lower-status workers who generally work in a supportive role to the higher-status occupation of veterinary professionals (31). Managers, supervisors and colleagues can provide positive feedback, support and respect one another in dealing with difficult client and animal situations and in doing so, can alleviate feelings of strain and burnout (50). Respect can foster a sense of teamwork and belonging to the workplace and may offer an important coping resource during times of stress. Since many veterinarians and veterinary technicians work in small work settings, they may not have regular interactions with other veterinary workers (53). The small size of many veterinary practices may necessitate that animal care workers seek out supportive ties and networks outside their own employment setting. Fortunately, many professional associations, such as NAVTA, are increasingly aware of the health care issues and needs of their members and offer well-being resources in various forms (e.g., online resources, peer assistances, help-lines).

In closing, it is important to highlight the potential efficacy of some no-cost interventions for combating burnout among veterinary technicians. While monetary limitations may preclude employers from sponsoring registration fees and offering paid time off for continuing education courses, recognizing employees' contributions toward providing excellent animal care incurs no cost. This recognition can go a long way toward reducing the cynicism elements of burnout and enhancing technicians' sense of professional efficacy. Likewise, creating a culture that destigmatizes the dirty work associated with veterinary technician positions can also be accomplished with little to no financial investment. The actual cost to the practice of allowing technicians' greater control over their own schedules depends on how the system is implemented. Possible solutions include allowing the existing technician staff to select their preferred shifts, or it could require hiring extra staff to cover the clinic during off-hours. Even though salary increases would undoubtedly be appreciated, the only correlation between financial rewards and burnout was a negative association between current pay/salary and the professional efficacy scale of MBI. This finding must be interpreted cautiously, as a causal order cannot be established.

Other limitations of this study include the cross-sectional methodology, as it only captured participants' feelings/perceptions at one point in time. While the sample size of 1,642 responses was large enough to provide statistical power, there is the potential for response bias because the response rate is equivalent to ~1.5% of the 109,000 currently employed veterinary technicians in the U.S. Finally, statistical correlation does not imply causation. Thus, we cannot conclusively state that the factors examined lead to burnout, but only that they are associated with higher burnout scores on the MBI and PFI.

Based on the findings of this study, future areas of research include the development and evaluation of technician recognition programs as a means to mitigate burnout. The relationship between salary and burnout should be explored in more detail. Finally, it might behoove veterinary practices to explore the cost of providing paid professional development opportunities as a means to enhance employee retention, especially in comparison to the cost of recruiting and training new hires.

## Data Availability Statement

The datasets generated for this study are available on request to the corresponding author.

## Ethics Statement

The studies involving human participants were reviewed and approved by Colorado State University Institutional Review Board (IRB # 086-19H). Written informed consent for participation was not required for this study in accordance with the national legislation and the institutional requirements.

## Author Contributions

LK and JW conceived the study. LK, JW, PH, and MR conducted the research. LK, JW, RS-T, and PH wrote the manuscript.

## Conflict of Interest

The authors declare that the research was conducted in the absence of any commercial or financial relationships that could be construed as a potential conflict of interest.
